# Estimation of Sensitive Proportion by Randomized Response Data in Successive Sampling

**DOI:** 10.1155/2015/172918

**Published:** 2015-05-18

**Authors:** Bo Yu, Zongda Jin, Jiayong Tian, Ge Gao

**Affiliations:** ^1^School of Public Health, Medical College, Soochow University, Suzhou 215123, China; ^2^School of Mathematical Sciences, Dezhou University, Dezhou 253023, China; ^3^Department of Public Health, Zhejiang Medical College, Hangzhou 310053, China; ^4^Critical Care Medicine, People's Hospital of Linshu County, Linyi, Shandong 276700, China

## Abstract

This paper considers the problem of estimation for binomial proportions of sensitive or stigmatizing attributes in the population of interest. Randomized response techniques are suggested for protecting the privacy of respondents and reducing the response bias while eliciting information on sensitive attributes. In many sensitive question surveys, the same population is often sampled repeatedly on each occasion. In this paper, we apply successive sampling scheme to improve the estimation of the sensitive proportion on current occasion.

## 1. Introduction

Social survey sometimes includes stigmatizing or sensitive issues of enquiry, such as habitual tax evasion, sexual behaviour, substance abuse, and excessive gambling that it is difficult to obtain valid and trustworthy information. If the respondents are asked directly about controversial matters, it often results in refusal or untruthful answers, especially when they have committed stigmatizing behaviour. To overcome this difficulty, Warner [1] introduced randomized response techniques to estimate the proportion of people bearing such a stigmatizing or sensitive characteristic in a given community. This technique allows the respondent to answer sensitive questions truthfully without revealing embarrassing behaviour. Following the pioneering work of Warner [1], some researchers have made important contributions in this area, such as Christofides [2, 3], Singh [4], Kim and Elam [5], Huang [6, 7], Singh and Sedory [8], Chang and Kuo [9], Arnab et al. [10]. All these results are based on a sample on one occasion, which is not the case in the present study.

In many sensitive question surveys, the same population is often sampled repeatedly on each occasion, so that the development over time can be followed. In such situations, the use of successive sampling scheme can be attractive alternative to improve the estimators of level at a point in time or to measure the change between two time points. In successive sampling on two occasions, previous theory [11, 12] aimed at providing the optimum estimator of mean on the current (second) occasion. Successive sampling has also been discussed in some detail by Narain [13], Raj [14], Singh [15], Ghangurde and Rao [16], Okafor [17], Arnab and Okafor [18], Biradar and Singh [19], G. N. Singh and V. K. Singh [20], Artes et al. [21], and so forth, and Singh et al. [22]. However no effort has been made to estimate the proportions of sensitive infinite population on the current occasion. This motivation led the authors to consider the problem of estimating the binomial proportions of sensitive or stigmatizing attributes in the population of interest in successive sampling on two occasions. In addition, cluster sampling is usually preferred when the target population is geographically diverse. In this paper, we utilize the rotation cluster sample design to construct a class of estimators for the case of randomized response survey. The rest of the paper is organized as follows. In Section 2, we proposed a new scientific survey method using the Simmons model with cluster rotation sampling. In Section 3, corresponding formulas for the mentioned survey method are found followed by the aforementioned method and corresponding formulas were successfully designed and applied in a survey of premarital sexual behaviour among students at Soochow University in Section 4.  Section 5 contains the conclusion.

## 2. The Proposed Survey Methods

### 2.1. Simmons Model

Simmons model which is based on Warner's randomized response technique was put forward by Horvitz et al. [23]. The basic thought is to develop a random rapport between the individuals and two unrelated questions. Simmons design consists of two unrelated questions, A and B, to be answered on probability basis, where A is “do you possess the sensitive characteristic” and B is a nonsensitive question such as “is your birthday number odd or not.” The two questions A and B are presented to respondents with preset probabilities *P* and 1 − *P*, respectively. The simple random sampling with replacement (SRSWR) is assumed. The selected respondent is asked to select a question A or B and report “yes” if his/her actual status matches with the selected question and “no” otherwise.

### 2.2. Simmons Model in Cluster Rotation Sampling

In the following sampling on two occasions is considered to estimate population proportion with a sensitive characteristic on second occasion when the rotation sampling units are clusters. The sampling steps for Simmons model under partial clusters rotation are as follows.

Firstly, the population is divided into primary sampling units (or cluster) and the units within the clusters are the secondary sampling units (persons).

Secondly, in the first occasion a random sample of *n* clusters with replacement is drawn from the population. The people within the drawn clusters are asked to select a question A or B and report “yes” if his/her actual status matches with the selected question and “no” otherwise, using the Simmons model.

Thirdly, in the second occasion *m* of the *n* clusters selected on the first occasion are retained at random and the remaining *u* = *n* − *m* of the clusters are replaced by a fresh selection. All the people within the total clusters in the second occasion are investigated using the Simmons model.

## 3. Formulas Deduction

### 3.1. The Estimator of the Population Proportion on the Second Occasion and Its Variance

Consider a random sample of *n* clusters with replacement drawn from the population which consists of *N* clusters and the *i*th cluster of *M*_*i*_ units (*i* = 1,2,…, *N*).

In the second (current) occasion *m* of the *n* clusters selected on the first occasion are retained at random and the remaining *u* = *n* − *m* of the clusters are replaced by a fresh selection. Let *a*_1,*l*_ be the number of the *l*th retained cluster (including *M*_*l*_ units) with the sensitive characteristic under study on the first occasion (*l* = 1,2,…, *m*) and let *a*_1,*m*+*k*_ be the number of the *k*th rotated cluster (including *M*_*m*+*k*_ units) with the sensitive characteristic under study on the first occasion, respectively (*k* = 1,2,…, *u*). *a*_2,*l*_ is the number of the *l*th retained cluster (including *M*_*l*_ units) with the sensitive characteristic under study on the second (current) occasion (*l* = 1,2,…, *m*) and *a*_2,*m*+*j*_ is the number of the *j*th fresh cluster (including *M*_*m*+*j*_′ units) with the sensitive characteristic under study on the second (current) occasion (*j* = 1,2,…, *u*). Similarly, let *π*_1,*l*_ be the proportion of the *l*th retained cluster with the sensitive characteristic under study on the first occasion (*l* = 1,2,…, *m*) and let *π*_1,*m*+*k*_ be the proportion of the *k*th rotated cluster with the sensitive characteristic under study on the first occasion (*k* = 1,2,…, *u*), respectively. *π*_2,*l*_ is the proportion of the *l*th retained cluster with the sensitive characteristic under study on the second (current) occasion (*l* = 1,2,…, *m*) and *π*_2,*m*+*j*_ is the proportion of the *j*th fresh cluster with the sensitive characteristic under study on the second (current) occasion (*j* = 1,2,…, *u*). Assume that the variance and the correlation coefficient between the first occasion and second occasion are constant s and the overall correction coefficient is ignored.

Define the following:

 
*π*_1_: the population proportion of the sensitive characteristic on the first occasion;
 
*π*_2_: the population proportion of the sensitive characteristic on the second occasion;
 
*π*_1__*m*_: the proportion of *m* retained clusters with the sensitive characteristic on the first occasion;
 
*π*_2__*m*_: the proportion of *m* retained clusters with the sensitive characteristic on the second occasion;
 
*π*_1__*u*_: the proportion of *u* rotated clusters with the sensitive characteristic on the first occasion;
 
*π*_2__*u*_: the proportion of *u* fresh clusters with the sensitive characteristic on the second occasion.

The following is according to the formula and results given by Cochran [24].

The estimator of *π*_1_ is

(1) $$\begin{array}{c} {\hat{\pi }}_{1}=\frac{\sum_{i=1}^{n}a_{1,i}}{\sum_{i=1}^{n}M_{i}}. \end{array}$$

The estimator of *π*_1*m*_ is

(2) $$\begin{array}{c} {\hat{\pi }}_{1m}=\frac{\sum_{i=1}^{m}a_{1,i}}{\sum_{i=1}^{m}M_{i}}. \end{array}$$

The estimator of *π*_1*u*_ is

(3) $$\begin{array}{c} {\hat{\pi }}_{1u}=\frac{\sum_{j=1}^{u}a_{1,m+j}}{\sum_{j=1}^{u}M_{m+j}\;}. \end{array}$$

The estimator of *π*_2*m*_ is

(4) $$\begin{array}{c} {\hat{\pi }}_{2m}=\frac{\sum_{j=1}^{m}a_{2,j}}{\sum_{j=1}^{m}M_{j}}. \end{array}$$

The estimator of *π*_2*u*_ is

(5) $$\begin{array}{c} {\hat{\pi }}_{2u}=\frac{\sum_{j=1}^{u}a_{2,m+j}}{\sum_{j=1}^{u}M_{m+j}^{\prime }}. \end{array}$$

Consider a generalized estimator ${\hat{\pi }}_{2}$ of the population proportion of the sensitive characteristic on the second occasion or current occasion as

(6) $$\begin{array}{c} {\hat{\pi }}_{2}=a{\hat{\pi }}_{1u}+b{\hat{\pi }}_{1m}+c{\hat{\pi }}_{2u}+d{\hat{\pi }}_{2m}, \end{array}$$

where *a*, *b*, *c*, and *d* are suitable constants.

We have

(7) $$\begin{array}{c} E\left({\hat{\pi }}_{2}\right)=\left(a+b\right)\pi_{1}+\left(c+d\right)\pi_{2}. \end{array}$$

Because the estimator ${\hat{\pi }}_{2}$  of *π*_2_ is an unbiased estimator of *π*_2_, we have *a* + *b* = 0 and *c* + *d* = 0.

Hence, the estimator 6 takes the form

(8) $$\begin{array}{c} {\hat{\pi }}_{2}=a\left({\hat{\pi }}_{1u}-{\hat{\pi }}_{1m}\right)+c{\hat{\pi }}_{2u}+\left(1-c\right){\hat{\pi }}_{2m}. \end{array}$$

The variance of estimator ${\hat{\pi }}_{2}$ is

(9) Var⁡π^2=a2Var⁡(π^1u)+a2Var⁡(π^1m)+c2Var⁡(π^2u) +1−c2Var⁡(π^2m)−2a(1−c)cov⁡(π^1m,π^2m)=a2S12u+a2S12m+c2S22u +1−c2S22m−2a1−cρS1S2m=a2S12u+S12m+c2S22u+1−c2S22m −2a1−cρbS1S2m.

Other covariance terms are zero.

Minimizing the variance of estimator ${\hat{\pi }}_{2}$ with respect to *a* and *c* when *N* is sufficiently large,

(10) $$\begin{array}{c} \frac{\partial \mathrm{Var}\left({\hat{\pi }}_{2}\right)}{\partial a}=2a\left(\frac{1}{u}+\frac{1}{m}\right)S_{1}^{2}-2\left(1-c\right)\frac{\rho_{b}S_{1}S_{2}}{m}=0,\\ \frac{\partial \mathrm{Var}\left({\hat{\pi }}_{2}\right)}{\partial c}=2c\left(\frac{S_{2}^{2}}{u}\right)-2\left(1-c\right)\left(\frac{S_{2}^{2}}{m}\right)+2a\left(\frac{\rho_{b}S_{1}S_{2}}{m}\right)=0. \end{array}$$

Then we get

(11) $$\begin{array}{c} a\left(\frac{1}{u}+\frac{1}{m}\right)S_{1}^{2}=\left(1-c\right)\frac{\rho_{b}S_{1}S_{2}}{m},\\ a\left(\frac{\rho_{b}S_{1}S_{2}}{m}\right)=c\left(\frac{S_{2}^{2}}{u}\right)-\left(1-c\right)\left(\frac{S_{2}^{2}}{m}\right). \end{array}$$

We derive

(12) $$\begin{array}{c} \frac{1/u+1/m}{\rho_{b}/m}=\frac{\left(1-c\right)\left(\rho_{b}/m\right)}{c/u-\left(1-c\right)/m}. \end{array}$$

One has

(13) $$\begin{array}{c} \frac{\left(u+m\right)/um}{\rho_{b}/m}=\frac{\left(1-c\right)\left(\rho_{b}/m\right)}{\left(c\left(u+m\right)-u\right)/um}, \end{array}$$

for *u* + *m* = *n*.

We have

(14) $$\begin{array}{c} \frac{n/u}{\rho_{b}}=\frac{\left(1-c\right)\rho_{b}}{\left(cn-u\right)/u}. \end{array}$$

Hence,

(15) $$\begin{array}{c} c=\frac{u/n-\left(u^{2}/n^{2}\right)\rho_{b}^{2}}{1-\left(u^{2}/n^{2}\right)\rho_{b}}. \end{array}$$

Define *μ* = *u*/*n*.

We get

(16) $$\begin{array}{c} c=\frac{\mu -\mu^{2}\rho_{b}^{2}}{1-\mu^{2}\rho_{b}^{2}}. \end{array}$$

By 16, we derive

(17) $$\begin{array}{c} a=\frac{\left(1-c\right)\left(\rho_{b}S_{2}\right)}{\left(m/u+1\right)S_{1}}, \end{array}$$

for *u* + *m* = *n* and *μ* = *u*/*n*.

One has

(18) $$\begin{array}{c} a=\frac{\left(1-c\right)\mu \left(\rho_{b}S_{2}\right)}{S_{1}}. \end{array}$$

By 16 and 18, we get

(19) $$\begin{array}{c} a=\frac{S_{2}}{S_{1}}\cdot \frac{\mu \left(1-\mu \right)\rho_{b}}{1-\mu^{2}{\rho_{b}}^{2}}, \end{array}$$

where

(20) $$\begin{array}{c} S_{h}^{2}=\frac{1}{N-1}\overset{N}{\underset{i=1}{\sum }}{\left(\pi_{h,i}-\pi_{h}\right)}^{2},\;h=1,2;\;\;\mu =\frac{u}{n},\\ \rho_{b}=\frac{\sum_{i=1}^{N}\left(\pi_{2,i}-\pi_{2}\right)\left(\pi_{1,i}-\pi_{1}\right)}{\sqrt{\sum_{i=1}^{N}{\left(\pi_{2,i}-\pi_{2}\right)}^{2}\sum_{i=1}^{N}{\left(\pi_{1,i}-\pi_{1}\right)}^{2}}}. \end{array}$$

**Theorem 1 .**

Under the Simmons model in partial clusters rotation, one has

(21) π^2=S2S1·μ1−μρb1−μ2ρb2(π^1u−π^1m)+μ−μ2ρb21−μ2ρb2π^2u+(1−μ1−μ2ρb2)π^2m,

and the variance of estimator ${\hat{\pi }}_{2}$ is

(22) Var⁡min⁡P^2=1−μρb21−μ2ρb2S22n.

**Remark 2 .**

In practice, the *ρ*_*b*_ and *S*_*h*_^2^ are unknown. The estimator of *ρ*_*b*_ is

(23) $$\begin{array}{c} {\hat{\rho }}_{b}=\frac{\sum_{k=1}^{m}\left(\pi_{2,k}-{\hat{\pi }}_{2m}\right)\left(\pi_{1,k}-{\hat{\pi }}_{1m}\right)}{\sqrt{\sum_{k=1}^{m}{\left(\pi_{2,k}-{\hat{\pi }}_{2m}\right)}^{2}\sum_{k=1}^{m}{\left(\pi_{1,k}-{\hat{\pi }}_{1m}\right)}^{2}}}. \end{array}$$

And the estimator of *S*_*h*_^2^ is

(24) sh2=1m−1∑i=1mπh,i−π^hm2, h=1,2.

**Theorem 3 .**

Under the Simmons model in partial clusters rotation, one has the optimum rotation rate as

(25) $$\begin{array}{c} \mu ={\left[1+\sqrt{1-{\rho_{b}}^{2}}\right]}^{-1}. \end{array}$$

And the optimum variance of estimator ${\hat{\pi }}_{2}$ is

(26) $$\begin{array}{c} {\mathrm{Var}}_{\min\left(\text{opt}\right)}\left({\hat{\pi }}_{2}\right)=\left[1+\sqrt{1-\rho_{b}^{2}}\right]\frac{S_{2}^{2}}{2n}. \end{array}$$

Practically, the costs of sample survey usually represent the following simple function, according to Cochran [24]:

(27) $$\begin{array}{c} C_{T}=c_{0}+c_{1}m+c_{2}u, \end{array}$$

where *C*_*T*_ is the total cost of sampling, *c*_0_ is the fundamental cost of the survey, *c*_1_ is the average fundamental cost of investigating one retained cluster on the second occasion, and *c*_2_ is the average fundamental cost of investigating one fresh cluster on the second occasion.

**Theorem 4 .**

Under the given cost of sample survey *C*_*T*_, one has

(28) $$\begin{array}{c} {\mathrm{Var}}_{\text{opt}}\left({\hat{\pi }}_{2}\right)=\frac{\left(c_{1}\sqrt{1-\rho_{b}^{2}}+c_{2}\right)S_{2}^{2}}{2\left(C_{T}-c_{0}\right)}. \end{array}$$

And the estimation of sample size in partial clusters rotation is

(29) $$\begin{array}{c} n^{\prime }=\frac{M\left(C_{T}-c_{0}\right)\left(1+\sqrt{1-\rho_{b}^{2}}\right)}{c_{1}\sqrt{1-\rho_{b}^{2}}+c_{2}}, \end{array}$$

where

(30) $$\begin{array}{c} S_{2}^{2}=\frac{1}{N-1}\overset{N}{\underset{i=1}{\sum }}{\left(\pi_{2,i}-\pi_{2}\right)}^{2}. \end{array}$$

### 3.2. The Estimator of *π*_*h*,*i*_

Let *R*_*h*,*i*_ be the proportion of the selected *i*th cluster (including *M*_*i*_ units) with the nonsensitive characteristic under study on the *h*th occasion; *λ*_*h*,*i*_ and *m*_*h*,*i*_ denote the number and the proportion of “yes” answers in the *i*th cluster, respectively, where ${\hat{\lambda }}_{h,i}=m_{h,i}/M_{i}$, *h* = 1,2, (*i* = 1,2,…, *n*).

From the total probability formulas (see [25]), we can get

(31) πh,i=λh,i−(1−P)Rh,iP,h=1,2, i=1,2,…,n.

Hence

(32) $$\begin{array}{c} a_{h,i}=M_{i}\pi_{h,i},\;h=1,2,\;\;i=1,2,\ldots ,n. \end{array}$$

## 4. Applications

### 4.1. Survey Design

The survey is about premarital sexual behavior among students in Dushu Lake Campus of Soochow University. We regard every class as a cluster of 45 persons per class on average. In the first occasion (2011), 12 classes were drawn from all the classes randomly. All the persons in the selected 12 classes are surveyed by Simmons model for sensitive questions. In the second occasion (2013), 8 of the 12 classes selected on the first occasion are retained at random and the remaining 4 classes are replaced by a fresh selection. Then all the persons in the selected classes that consist of 8 retained classes and 4 fresh classes are surveyed by Simmons model for sensitive questions.

In our design, each person was asked to draw a ball at random with replacement from a bag containing 6 red balls and 4 white balls with known probability (the proportion of red balls was 0.6). If a red ball was selected by the respondent, then he or she would be asked the sensitive question A, where A is “are you a member of the group having premarital sexual behavior.” If a white ball was selected, he or she would answer the nonsensitive question B, where B is “is your student number odd or not.” The respondent reports “yes” if his/her actual status matches with the selected question and “no” otherwise.

All the questionnaires of two occasions had been checked to ensure that they are completed independently and no questions were omitted. The recovery rate of the survey was 100% with no failure questionnaire. All data was processed and analyzed by Excel 2003 and SAS 9.13.

### 4.2. Results

#### 4.2.1. Result of the Survey

In our design, each person was asked to draw a ball at random with replacement from a bag containing 6 red balls and 4 white balls with known probability (the proportion of red balls was 0.6). If a red ball was selected by the respondent, then he or she would be asked the sensitive question A, where A is “are you a member of the group having premarital sexual behavior.” If a white ball was selected, he or she would answer the nonsensitive question B, where B is “is your student number odd or not.” The respondent reports “*yes*” if his/her actual status matches with the selected question and “*no*” otherwise. According to 31, we get the sample proportion of the undergraduate students who have premarital sexual behavior *π*_*h*,*i*_, *h* = 1,2, as is shown in Table 1.

#### 4.2.2. The Estimator of the Population Proportion on the Second Occasion and Its Variance

By 1, the estimator of the population proportion with premarital sexual behavior on the first occasion is as follows: ${\hat{\pi }}_{1}=0.142933$.

According to 24, 2, and 3, we have

(33) $$\begin{array}{c} s_{1}^{2}=0.005853,\;{\hat{\pi }}_{1m}=0.1458,\;{\hat{\pi }}_{1u}=0.1372, \end{array}$$

respectively.

According to the results of investigation premarital sexual behavior among students in Dushu Lake Campus of Soochow University on the second occasion, from formulae 4 and 5,

(34) $$\begin{array}{c} {\hat{\pi }}_{2m}=0.1562,\;\;{\hat{\pi }}_{2u}=0.1783. \end{array}$$

By 23 and 24, we obtain *S*_2_^2^ = 0.004262 and ${\hat{\rho }}_{b}=0.936$, respectively.

From formula 25, *μ* = 0.7985; then according to formula 21, we get

(35) π^2=0.2912(0.1372−0.1458)+0.5453×0.1783+(1−0.5453)×0.1562=0.1657.

Using 22, we get $\text{Va}{\text{r}}_{\min(\text{opt})}({\hat{\pi }}_{2})=0.00024$. Hence, standard deviation is as follows:

(36) $$\begin{array}{c} \sqrt{\text{Va}{\text{r}}_{\min(\text{opt})}\left({\hat{\pi }}_{2}\right)}=0.01549. \end{array}$$

So, 95% confidence interval of the population proportion with the premarital sexual is [0.1353,0.1961].

## 5. Discussion and Conclusion

To sum up, in this study, we proposed a new sampling method to solve the question of sensitive questions surveys repeated over time, which is the first attempt made by the authors in this direction. Then the corresponding formulas for the estimator of the population proportion with sensitive characteristic and its variance for the proposed sampling method are provided. In addition, formulas for the optimal rotation rate and sample size under the given cost of sample survey are given.

The aforementioned method and corresponding formulas were successfully designed and applied in the premarital sex survey in Dushu Lake Campus of Soochow University. In a word, the designed sampling method and corresponding formulas have important theory and application value to achieve the sensitive questions continuous survey.

## 6. Proofs of Theorems

**Proof of Theorem 1.**

Using the optimum values of *a* and *c* given by 16 and 19, estimator ${\hat{\pi }}_{2}$ reduces to 21.

By 9, 16, and 19, we have

(37) $$\begin{array}{c} \text{Va}{\text{r}}_{\min}\left({\hat{P}}_{2}\right)=\left[\frac{1-\mu {\rho_{b}}^{2}}{1-\mu^{2}{\rho_{b}}^{2}}\right]\frac{S_{2}^{2}}{n}. \end{array}$$

**Proof of Theorem 3.**

The optimum value of *μ* is given by further minimizing 22 with respect to *μ*,

(38) $$\begin{array}{c} \frac{\partial \text{Va}{\text{r}}_{\min}\left({\hat{\pi }}_{2}\right)}{\partial \mu }=0. \end{array}$$

So

(39) $$\begin{array}{c} \mu ={\left[1+\sqrt{1-\rho_{b}^{2}}\right]}^{-1}. \end{array}$$

Substituting 39 in 22, we have the optimum variance of estimator ${\hat{\pi }}_{2}$ as

(40) $$\begin{array}{c} \text{Va}{\text{r}}_{\min\left(\text{opt}\right)}\left({\hat{\pi }}_{2}\right)=\left[1+\sqrt{1-\rho_{b}^{2}}\right]\frac{S_{2}^{2}}{2n}. \end{array}$$

**Proof of Theorem 4.**

ByTheorem 3,

(41) $$\begin{array}{c} \mu ={\left[1+\sqrt{1-\rho_{b}^{2}}\right]}^{-1},\;\mu =\frac{u}{n},\;\;u=n-m. \end{array}$$

Substituting 41 in 27, we obtain

(42) $$\begin{array}{c} n=\frac{\left(C_{T}-c_{0}\right)\left(1+\sqrt{1-\rho_{b}^{2}}\right)}{c_{1}\sqrt{1-\rho_{b}^{2}}+c_{2}}. \end{array}$$

Suppose the average cluster consists of $\overset{-}{M}$ units; then

(43) $$\begin{array}{c} n^{\prime }=\frac{\overset{-}{M}\left(C_{T}-c_{0}\right)\left(1+\sqrt{1-\rho_{b}^{2}}\right)}{c_{1}\sqrt{1-\rho_{b}^{2}}+c_{2}}. \end{array}$$

Substituting 42 in 26, we have

(44) $$\begin{array}{c} \text{Va}{\text{r}}_{\text{opt}}\left({\hat{\pi }}_{2}\right)=\frac{\left(c_{1}\sqrt{1-\rho_{b}^{2}}+c_{2}\right)S_{2}^{2}}{2\left(C_{T}-c_{0}\right)}. \end{array}$$

## Figures and Tables

**Table 1:** The proportion of every class with premarital sexual behavior in first and second occasions.

Class number	π_1,*i*_	π_2,*i*_
1	0.2624	0.2348
2	0.1631	0.1945
3	0.2101	0.2264
4	0.2063	
5	0.1556	0.1986
6	0.2390	
7	0.1783	
8	0.1970	0.1550
9	0.0123	0.0114
10	0.0476	0.0738
11	0.0455	
12	0.1185	0.1187
13		0.2035
14		0.1587
15		0.1926
16		0.1583
